# Concerning Memorials

**Published:** 1924-08

**Authors:** Theodore Ruete


### CONCERNING MEMORIALS.

By THEODORE RUETE.

In most parts of the world, so far back as human history can be traced, the custom has prevailed of erecting memorials to the departed. It still persists among us, and always will persist, the human heart being what it is. Only the manner of it changes according to the vision of those practising it, and the materials obtainable in any particular spot. People in time came to look upon memorials from a more spiritual standpoint, and began to realise that a benefaction affecting future generations would serve to keep alive the remembrance of the absent as well as, if not better than, any piece of bronze or marble.

### A Great Idea.

The best of all memorials to the dead are those established to rest and refresh the poor and weary, and to prevent them from falling sick. Of such are fresh-air funds, holiday camps and similar enterprises. It is, indeed, for the increase of monuments like these, erected not on country sward or city pavement, but in the hearts and lives of fellow-men, that we modern people must labour if we would claim to possess any real vision of immortality. Looked at, then, from this standpoint, the glorified Army hut, set up in the large garden and orchard of a fruit-grower near Longfield in Kent, in remembrance of two dearly loved sons, seems, in its practical service and vivid appeal to our imagination, to be an ideal private memorial. It is true that it is made of galvanised iron, which is not exactly an ideal material ; but, carefully kept in order for needy friends and tired helpers of the poor, it is likely to last a considerable time. Settlement workers, missioners, nurses, deaconesses, lay readers and the many gently-bred, whose fixed incomes yet place them nowadays among the new-poor, will find in this a hut, the erection and fitting of which were evidently a labour of love. This roomy little building is painted outside with alternate broad stripes of red and black, and inside is all enamel white. A comfortable, double brass bed, easy chairs, dressing-table, camp washstand, table, large cupboard and a double-burner oil-stove with oven make the place comfortable and convenient. Thoughtful care for the welfare of its occupants, too, is shown by the provision of pots and pans, soap, matches, magazines and even postage stamps.

### A Scheme to be Copied.

In the cool cellar in the chalk, close behind the hut, a neat meat-safe, alongside that of the family, accommodates the provisions of the visitors, which—supplemented by fresh eggs, fruit and vegetables from the homestead—they buy and personally cook. The occupants of the hut can thus freely roam about the orchard and the glorious country in this " garden of England," while its owners suffer naught from their constant succession of guests, of whom they can see much or little, as they choose. This original and most worthy memorial is booked up to the end of September, and the scheme might well be copied by others.

